# Differential Reflecting Frequency Modulation with QAM for RIS-Based Communications

**DOI:** 10.3390/s26030802

**Published:** 2026-01-25

**Authors:** Yajun Fan, Le Zhao, Wencai Yan, Haihua Ma

**Affiliations:** 1 The Key Laboratory of Grain Information Processing and Control (Henan University of Technology), Ministry of Education, Zhengzhou 450001, China; yjfan@haut.edu.cn (Y.F.); lezhao@haut.edu.cn (L.Z.); mahaihua@haut.edu.cn (H.M.); 2 The College of Information Science and Engineering, Henan University of Technology, Zhengzhou 450001, China

**Keywords:** differential reflecting frequency modulation, quadrature amplitude modulation, reconfigurable intelligent surface

## Abstract

Reconfigurable intelligent surface (RIS)-aided index modulation (IM) shows great potential for next-generation wireless communications. Nevertheless, obtaining channel state information (CSI) for RIS-based IM incurs high pilot overhead, particularly for multi-domain IM. In this paper, we integrate orthogonal frequency division multiplexing into RIS-aided differential reflecting modulation (DRM) communications, introducing the differential reflecting frequency modulation (DRFM) system. In DRFM, information bits are jointly conveyed through the activation permutations of reflecting patterns, grouped carriers, and constellation symbols. The transmitter combines the differentially coded reflecting-time block and the time–frequency block using the Kronecker product. This allows DRFM to operate without relying on CSI at the transmitter, RIS, or receiver. Moreover, we design a novel high-rate quadrature amplitude modulation (QAM) scheme for DRFM. Compared to PSK-based DRFM, this QAM scheme can boost either the throughput or the performance of DRFM. Simulation results illustrate the superiority of the DRFM system, along with an acceptable SNR penalty, compared to non-differential modulation with coherent detection. At the same spectral efficiency, the proposed QAM-aided DRFM outperforms schemes using traditional PSK, amplitude phase shift keying (APSK), and star-QAM constellation modulations.

## 1. Introduction

### 1.1. Background

Owing to its advantages in energy efficiency and error performance, index modulation (IM) is a promising technology for sixth-generation wireless communications [1,2,3,4]. The IM technique conveys additional bits via common resources such as antennas [5,6,7], frequencies [8,9], timeslots [10], codes [11], and beamspace. As another emerging IM technique, reconfigurable intelligent surface (RIS)-based reflection modulation (RM) regards different reflection patterns of the RIS as the index entities of IM signals [12,13]. The RIS consists of a substantial number of low-cost passive reflective elements. These elements, with the assistance of an intelligent controller, are capable of modulating the phase shift and amplitude gain of the passive reflection of incident electromagnetic waves [14]. By leveraging the dimension of reflection patterns, the RM technology enhances the channel gain of the communication system.

However, the receivers in all of the aforementioned schemes focus on IM for coherent communications, in which highly precise channel state information (CSI) is of the utmost necessity for signal detection [15,16,17]. Whether in traditional multiple-input multiple-output (MIMO) wireless communication systems or in RIS-assisted MIMO systems, channel estimation is a challenging and resource-intensive task. Additionally, the performance degradation resulting from channel estimation errors is unavoidable. To circumvent complex channel estimation, a commonly adopted approach is to employ differential modulation [18] for the joint IM entity across diverse domains. Leveraging the time dimension, differential spatial modulation (DSM), a representative non-coherent modulation scheme in the spatial domain, encodes a portion of the information bits within the activated antenna index matrices of a space-time block. By means of differential processing implemented at the transmitter, coupled with the corresponding non-coherent detection strategies employed at the receiver, DSM eliminates the requirement for highly accurate CSI and further reduces the complexity.

Inspired by DSM, the differential reflecting modulation (DRM) scheme was introduced for RIS-based communication systems [19,20], where additional information bits are conveyed via the activation permutation of the reflecting patterns. To further augment the beamforming gain of RIS, a method integrating RIS beam training and differential phase shift keying modulation at the transmitter was proposed [21]. Additionally, to eliminate the requirement for synchronizing the transmitted bits at both the transmitter and the RIS, an RIS-assisted differential transmitted spatial modulation was put forward [22], where the DSM-encoding process is implemented at the transmit antennas, with a single antenna activated per timeslot. Although the aforementioned RIS-based communication schemes [19,21] can avoid resource-consuming channel estimation, they operate solely within a single domain of IM. Consequently, this leads to low transmission efficiency.

### 1.2. Related Works and Motivations

In terms of boosting the transmission efficiency, very few existing studies [23,24,25,26] have focused on dual-domain differential modulation for RIS-enabled communication systems. Specifically, in [23], two differential schemes, namely DRM-aided spatial modulation and DSM-aided reflecting modulation, were presented, which are designed for joint differential IM in both the space and reflection domains [24]. To further enhance the bit error rate performance and decrease the decoding complexity, the authors of [25] proposed a multi-domain joint rectangular differential IM system that utilizes RF mirrors. This scheme devises a space media precoding codebook, which serves to jointly regulate the activation patterns of RF mirrors and antenna activation, enabling more efficient mapping within the space media domain. Similarly, the authors of [26] put forward the rectangular differential reflecting spatial modulation system, where a rectangular dispersion matrix is utilized to jointly map a digital beamforming weight vector and an RIS reflection pattern, thereby performing rectangular differential modulation. However, the studied RIS-based differential communication systems do not take frequency-domain resources into account. To address such limitations, we focus on differential reflecting frequency modulation (DRFM) for the RIS-aided communication system.

It is well known that, in most existing research on differential modulation systems, constant-modulus constellations—for instance, phase shift keying (PSK)—are adopted at the differential encoder [18]. Nevertheless, choosing such a constellation results in either reduced throughput or deteriorated error rate performance because of the small minimum distance in PSK. To increase the minimum distance while maintaining the constellation size, the authors of [27] put forward two-ring amplitude phase shift keying (APSK) modulation for the DSM system. A power level change coefficient normalizes the transmit signal power during differential processing between the rings of the APSK constellation. It has been shown that APSK-modulated DSM systems improve the bit error rate (BER) performance relative to conventional PSK-modulated DSM. Analogously, the authors of [28] presented a multi-ring amplitude phase modulation scheme. Through an in-depth analysis of the average bit error probability, the ratio of rings within the multi-amplitude constellation was optimized, which led to a further enhancement in the BER performance in the DSM system. In [29], a generalized transmission scheme, namely the precoding-normalized DSM scheme, was proposed for constant-modulus constellation modulation. Through normalizing the power of all symbols in the preceding transmit matrix during differential transmission, the BER performance was further improved. Different from the above modulation schemes, the authors of [30] proposed a quadrature amplitude modulation (QAM) method and applied it to the DSM system. The transmitted QAM constellation is encoded and transmitted in a hierarchical differential manner through the composite phase shift method. Inspired by the above idea, a non-constant QAM constellation modulation scheme is studied for the proposed DRFM system in this work.

### 1.3. Contributions

Compared with existing work, the main contributions of this study are summarized as follows:

- DRM is generalized from single-carrier to multi-carrier scenarios for RIS-based communications. In DRFM, information bits are jointly conveyed through the activation permutations of reflecting patterns, grouped carriers, and constellation symbols.
- An overall DRFM transceiver design is presented. The transmitter integrates the differentially coded reflecting-time block and the time–frequency block using the Kronecker product. At the receiver, differential detection allows operation without acquiring CSI.
- A non-constant QAM constellation modulation scheme is studied for the proposed DRFM system. The transmitted QAM constellation is encoded and transmitted in a hierarchical differential manner through the composite phase shift method.

### 1.4. Organization and Notations

The remainder of this paper is organized as follows. Section 2 describes the PSK-aided DRFM system, including the system description and transceiver design. Section 3 details the QAM-aided DRFM system, covering the transmitter design and receiver detection. Simulations are presented in Section 4, and Section 5 concludes the paper.

*Notations*: A, a and *a* stand for a matrix, a vector and a scalar, respectively. $A^{T}$ denotes the transpose operator of a vector. ${∥\cdot ∥}_{F}$ represents the Frobenius norm of a vector. $diag\left\{•\right\}$ indicates that a row vector is transformed into a diagonal matrix. $\left\lfloor •\right\rfloor$ stands for the floor operation. mod(a,b) denotes the modulo operation. The X×X identity matrix is represented by $I_{X}$. The complete list of acronyms is presented in Table 1.

## 2. PSK-Aided DRFM System

### 2.1. System Description

**Figure 1:**
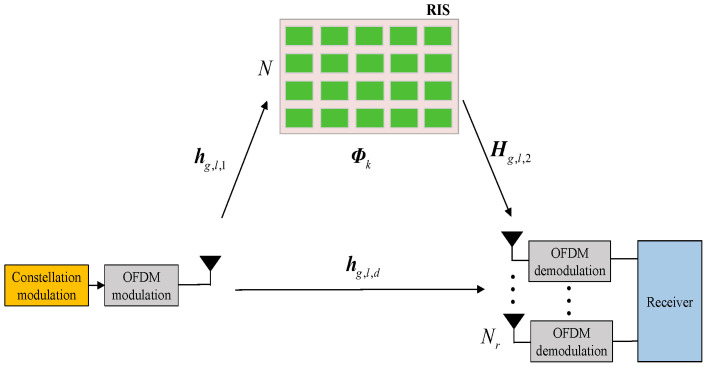
An RIS-based SIMO OFDM communication system.

In this work, we consider an RIS-based single-input multiple-output (SIMO) orthogonal frequency division multiplexing (OFDM) communication system model, as illustrated in Figure 1. $N_{r}$ represents the number of receiver (Rx) antennas, and *N* denotes the number of RIS reflecting units. Given the differential encoding mechanism at the transmitter (Tx), in this section, the Tx is tasked with sending *M*-ary phase shift keying (PSK) symbols.

Assume that the OFDM modulator is composed of *F* subcarriers, which are partitioned into *G* groups. Each group *g* contains L=F/G subcarriers. For group *g*, subcarrier *l*, let $h_{g,l,1}\in C^{N\times 1}$, $H_{g,l,2}\in C^{N_{r}\times N}$, and $h_{g,l,d}\in C^{N_{r}\times 1}$ stand for the channel vector between the RIS and the Tx, the channel matrix between the Rx and the RIS, and the channel vector between the Rx and the Tx, respectively. The channels are modeled as quasi-static Rayleigh fading channels, similarly to [19]. Unlike conventional RIS-aided systems, where the RIS passively improves the channel conditions, the proposed DRFM scheme brings about a paradigm shift. In this new approach, the RIS itself functions as an active information modulator in the spatial domain. This forms the fundamental reflecting dimension of our index modulation. Specifically, a predefined codebook consisting of *K* distinct reflection patterns is defined as $\Psi =\{\Phi_{1},\Phi_{2},\ldots ,\Phi_{K}\}$. Each candidate pattern $\Phi_{k}\in C^{N\times N}\left(k=1,2,\ldots ,K\right)$ is a diagonal matrix. The *n*-th diagonal entry ${\left(\Phi_{k}\right)}_{nn}$ satisfies $|{\left(\Phi_{k}\right)}_{nn}|=1$ for an ideal phase-shifting element or 0 to represent an inactive element, with a phase shift θ∈[0,2π). During the transmission process, a subset of these patterns is actively selected over time in accordance with the input data bits. In this way, information is directly embedded into the wireless channel through controlled reflections. This dynamic, information-driven reconfiguration of the RIS represents the physical implementation of reflecting modulation in DRFM.

### 2.2. Transceiver Design

#### 2.2.1. Differential Encoding Scheme

**Figure 2:**
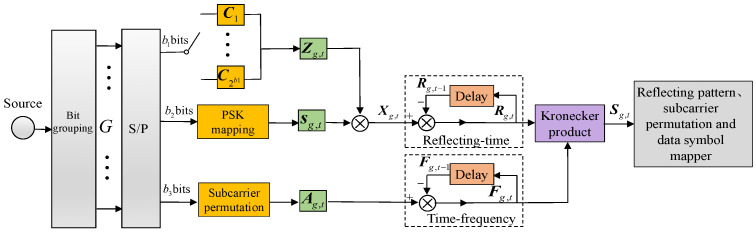
PSK-aided DRFM encoding process.

At the Tx, the original input bit stream is divided into *G* branches. Each branch comprises $B=b_{1}+b_{2}+b_{3}$ bits. During transmission, a block will occupy *L* symbol timeslots, such that L=K. As shown in Figure 2, at group *g*, time instant *t*, the first $b_{1}=\left\lfloor \log_{2}\left(K!\right)\right\rfloor$ bits are used to select the reflecting-time permutation matrix $Z_{g,t}$. Similarly to DSM, there are K! valid K×K permutation matrices, namely $C_{1},C_{2},\ldots ,C_{K!}$. In the mapping process, however, only $2^{b_{1}}$ matrices are chosen for the mapping operation. The other $b_{2}=K\log_{2}\left(M\right)$ are mapped into *K M*-ary PSK symbols $s_{g,t}={\left[s_{g,1},s_{g,2},\ldots ,s_{g,K}\right]}^{T}$. Finally, the rest $b_{3}=\left\lfloor \log_{2}\left(L!\right)\right\rfloor$ are used to select the time–frequency permutation matrix $A_{g,t}$. Assume that, over *L* subcarriers, the permutation of the activated reflecting patterns in *K* time instants stays constant. Under this premise, according to [9], the spectral efficiency of DRFM can be expressed as

(1) $$\begin{array}{c} \eta_{\mathrm{DRFM}}=\frac{G(\log_{2}\left(L!\right)+\log_{2}\left(K!\right)+K\log_{2}\left(M\right))}{K(F+L_{cp})} \end{array}$$

where $L_{cp}$ is the length of the cyclic prefix added at the Tx.

Assume that the Tx and the RIS can achieve good synchronization; the reflecting-time block that contains both the reflection pattern and symbol information can be expressed as

(2) $$\begin{array}{c} X_{g,t}=\mathrm{diag}\left\{s_{g,t}\right\}Z_{g,t} \end{array}$$

Based on differential encoding, the new time–frequency matrix and reflecting-time matrix are separately generated as

(3) $$\begin{array}{cc} \; & F_{g,t}=F_{g,t-1}A_{g,t} \end{array}$$

(4) $$\begin{array}{cc} \; & R_{g,t}=R_{g,t-1}X_{g,t} \end{array}$$

where $F_{g,t-1}$ and $R_{g,t-1}$, respectively, represent the time–frequency matrix and the reflecting-time matrix of group *g* at the previous time instant. At group *g*, time instant *t*, a non-zero element located at the (v,w) position of $F_{g,t}$ indicates that subcarrier *n* at timeslot *w* is activated to convey information. Similarly, a non-zero element at the (m,w) position of $R_{g,t}$ signifies that reflection pattern *m* at timeslot *w* is activated to transmit the modulated symbol $f_{mw}$. When t=0, $F_{g,0}=I_{X}$ and $R_{g,0}=I_{K}$ are designated as initial matrices. By leveraging the Kronecker product [9] to integrate the three-dimensional information of the reflection pattern, time, and frequency, the transmitted two-dimensional matrix $S_{g,t}\in C^{LK\times LK}$ can be formulated as

(5) $$\begin{array}{c} S_{g,t}=F_{g,t}⊗R_{g,t} \end{array}$$

Assume that $\psi_{F}$ and $\psi_{R}$, respectively, denote the sets encompassing all possible forms of F and R in Equation (5). For DRFM, it is necessary to satisfy the following: (1) given that $A_{g,t}\in \psi_{F}$ and $F_{g,t-1}\in \psi_{F}$, then $F_{g,t}\in \psi_{F}$; (2) given that $X_{g,t}\in \psi_{R}$ and $R_{g,t-1}\in \psi_{R}$, then $R_{g,t}\in \psi_{R}$.

#### 2.2.2. Channel Model

According to [9], at group *g*, the frequency-domain channel coefficient matrix can be expressed as

(6) $$\begin{array}{c} H_{g}=\mathrm{diag}{\left\{H_{g,l}\right\}}_{l=1}^{L} \end{array}$$

where $H_{g,l}={\tilde{H}}_{g,l,d}+{\tilde{H}}_{g,l,2}Q{\tilde{H}}_{g,l,1}\in C^{N_{r}\times K}$ represents the frequency-domain channel matrix of subcarrier *l* in group *g*. Specifically, according to [19], the matrices ${\tilde{H}}_{g,l,d}$, ${\tilde{H}}_{g,l,2}$, Q, and ${\tilde{H}}_{g,l,1}$ are, respectively, set as

(7) $$\begin{array}{cc} \; & {\tilde{H}}_{g,l,d}=\overset{K}{\overset{︷}{\left[h_{g,l,d},h_{g,l,d},\ldots ,h_{g,l,d}\right]}}\in C^{N_{r}\times K} \end{array}$$

(8) $$\begin{array}{cc} \; & {\tilde{H}}_{g,l,2}=\overset{K}{\overset{︷}{\left[H_{g,l,2},H_{g,l,2},\ldots ,H_{g,l,2}\right]}}\in C^{N_{r}\times KN} \end{array}$$

(9) $$\begin{array}{cc} \; & Q=\left[\begin{array}{cccc} \Phi_{1} & 0 & \ldots & 0\\ 0 & \Phi_{2} & \ldots & 0\\ \vdots & \vdots & \ddots & \vdots \\ 0 & 0 & \ldots & \Phi_{K} \end{array}\right]\in C^{KN\times KN} \end{array}$$

(10) $$\begin{array}{cc} \; & {\tilde{H}}_{g,l,1}=\overset{K}{\overset{︷}{\left[\begin{array}{cccc} h_{g,l,1} & 0 & \ldots & 0\\ 0 & h_{g,l,1} & \ldots & 0\\ \vdots & \vdots & \ddots & \vdots \\ 0 & 0 & \ldots & h_{g,l,1} \end{array}\right]}}\in C^{KN\times K} \end{array}$$

where Equations (7)–(10) formulate the aggregated channel matrices $H_{g,l}$ for subsequent derivations. In particular, Equation (7) aligns the direct path with each candidate RIS pattern. Equations (8) and (10) expand the RIS–receiver and RIS–transmitter channels, respectively, to match the dimensions of the RIS pattern set. Equation (9) encompasses all *K* possible reflection patterns from which the transmission patterns need to be selected within a finite set. Given that neither the transceivers nor the RIS have access to the CSI, we adopt the optimization criterion put forward in [19]. The objective of this criterion is to maximize the minimum mutual Euclidean distance, thereby enabling the derivation of all *K* reflection patterns. The mentioned formulations offer a concise foundation for the system model presented in Equation (11). Furthermore, although RIS commonly assists blocked links, the direct path $h_{g,l,d}$ is included to maintain model generality. It can be set to zero when the direct path is not present.

#### 2.2.3. ML Detector

At the Rx, the received matrix at group *g*, time instant *t* can be expressed as

(11) $$\begin{array}{c} Y_{g,t}=H_{g}S_{g,t}+V_{g,t} \end{array}$$

where $V_{g,t}\in C^{N_{r}L\times KL}$ denotes the complex additive white Gaussian noise matrix with zero mean and $\sigma^{2}1_{N_{r}X\times KX}$.

Substituting Equations (3)–(5) into Equation (11) yields

(12) $$\begin{array}{c} Y_{g,t}=H_{g}\left(F_{g,t-1}A_{g,t}\right)⊗\left(R_{g,t-1}X_{g,t}\right)+V_{g,t} \end{array}$$

In the aforementioned equation, we simplify it by leveraging the “mixed-product property” of the matrix product and Kronecker product. Specifically, given that A, B, C, and D are four matrices such that the matrix products AC and BD are well defined, the property ((A⊗B)(C⊗D)=(AC)⊗(BD)) holds. By virtue of this property and Equation (11), Equation (12) can be transformed into

(13) $$\begin{array}{cc} Y_{g,t} & =H_{g}\left(F_{g,t-1}⊗R_{g,t-1}\right)\left(A_{g,t}⊗X_{g,t}\right)+V_{g,t}\\ \; & =Y_{g,t-1}\left(A_{g,t}⊗X_{g,t}\right)+W_{g,t} \end{array}$$

where $W_{g,t}=V_{g,t}-V_{g,t-1}\left(A_{g,t}⊗X_{g,t}\right)$ denotes the received noise of the Rx at group *g*, time instant *t*.

Consequently, the optimal maximum-likelihood (ML) detector can function without any knowledge of CSI by

(14) $$\begin{array}{c} \left\{{\hat{A}}_{g,t},{\hat{X}}_{g,t}\right\}=\underset{\forall X_{g}\in \psi_{R},\forall A_{g}\in \psi_{F}}{\arg\min}{∥Y_{g,t}-Y_{g,t-1}\left(A_{g}⊗X_{g}\right)∥}_{F}^{2} \end{array}$$

where $\psi_{R}$ and $\psi_{F}$ are the sets of all legitimate $X_{g}$ and $A_{g}$, respectively. Equation (14) is solved by a joint exhaustive search over all valid combinations of the reflecting pattern matrix $A_{g}$ from $\psi_{F}$ and the symbol matrix $X_{g}$ from $\psi_{R}$. The pair that minimizes the Frobenius norm metric $∥Y_{g,t}-Y_{g,t-1}\left(A_{g}⊗X_{g}\right){∥}_{F}^{2}$ is selected. Decoding involves three parallel streams of bit recovery corresponding to the three bit partitions, i.e., $b_{1}$, $b_{3}$, and $b_{3}$. First, the time–frequency permutation $b_{3}$ bits are directly recovered by performing a codebook lookup on the estimated matrix ${\hat{A}}_{g,t}$. The core of decoding lies in the decomposition of the reflecting-time block, defined as $X_{g,t}=\mathrm{diag}\left(s_{g,t}\right)Z_{g,t}$, where $Z_{g,t}$ is a permutation matrix. Crucially, the specific permutation pattern of the matrix ${\hat{Z}}_{g,t}$ can be directly identified from the unique position of the non-zero element in each row of the estimated ${\hat{X}}_{g,t}$. This allows the receiver to unambiguously determine which predefined permutation matrix was transmitted. Once ${\hat{Z}}_{g,t}$ is known, the estimated constellation symbol vector ${\hat{s}}_{g,t}$ is obtained by applying the inverse permutation ${\hat{Z}}_{g,t}^{T}$ to the vector of non-zero elements extracted from ${\hat{X}}_{g,t}$. Finally, the remaining bits are recovered. The identified matrix ${\hat{Z}}_{g,t}$ undergoes a second codebook lookup to recover the $b_{1}$ reflection pattern index bits. Simultaneously, the $b_{2}$ constellation bits are demapped. For PSK, ${\hat{s}}_{g,t}$ is processed by a standard PSK demapper.

## 3. QAM-Aided DRFM System

As is well known, a differential modulation system demands a differential coding mechanism at the transmitter. Thus, in Section 2, we put forward a PSK-based DRFM system to circumvent amplitude accumulation problems in the differential process. For high-order modulation, compared to non-constant-modulus modulations like QAM, PSK modulation has a smaller minimum Euclidean distance between constellation points. This results in the relatively poor bit error performance of the differential system. To tackle this issue, we propose conducting the hierarchical transmission of the QAM constellation. In each layer, PSK is utilized for differential coding and transmission. This approach serves to prevent the issue of amplitude accumulation during the transmission of the QAM constellation. To further mitigate error propagation effects, we introduce QAM into the DRFM system.

### 3.1. Transmitter Design

**Figure 3:**
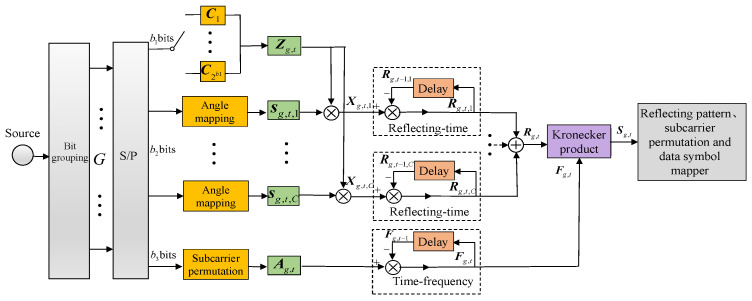
QAM-aided DRFM encoding process.

**Figure 4:**
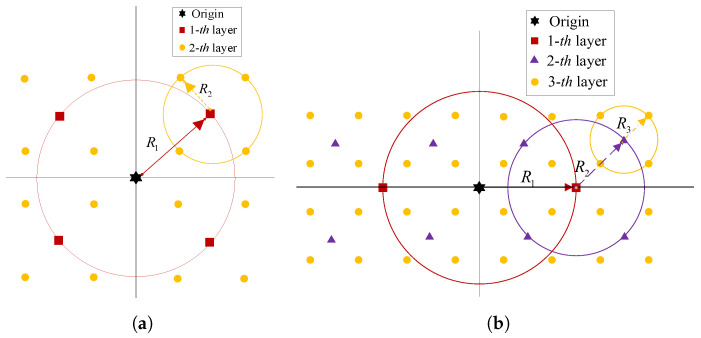
QAM constellation. (**a**) 16-QAM (*C* = 2). (**b**) 32-QAM (*C* = 3).

A block diagram of the proposed QAM-aided DRFM transmitter is given in Figure 3. Similarly to the PSK-aided Tx mapping scheme in Section 2, $b_{1}$ bits and $b_{3}$ bits are employed to select the reflecting-time permutation matrix $Z_{g,t}$ and the time–frequency permutation matrix $A_{g,t}$, respectively. The difference is that the $b_{2}=K\log_{2}\left(M\right)$ bits are mapped into *K M*-ary QAM symbols ($M=2^{m}$, m≥3). Figure 4 depicts the QAM constellation diagrams of various orders. Specifically, Figure 4a shows 16-QAM and Figure 4b represents 32-QAM. Here, C=⌊(m+1)/2⌋ denotes the total number of superimposed layers. Evidently, each QAM symbol is formed by superimposing multiple independent layers of BPSK or QPSK symbols. From Figure 4a, the 16-QAM modulation symbol is obtained by vectorially adding the first-layer QPSK vector (the four square points on the red circle) and the second-layer QPSK vector (the four dot points on the yellow circle). The bit mapping of modulation symbols in each layer is accomplished via distinct angle mappings. The angular vector of the *c*-th layer’s modulation symbols can be denoted as $\theta_{g,t,c}=\left[\theta_{g,t,c,1},\ldots ,\theta_{g,t,c,K}\right]$(c=1,2,…,C). If *m* is odd, the first-layer angular mapping rule is as follows: when the one bit a=0, the mapping results in θ=0; when a=1, the mapping gives θ=π. When *m* is even, the first-layer angular mapping rule is as follows: when the two bits $a_{1}a_{2}=00,01,10$, and 11, the mapping results in θ=0,π/2,π and 3π/2, respectively.

Based on the angle mapping, the transmission signal vector $s_{g,t,c}$ of layer *c* is obtained as $s_{g,t,c}={\left[e^{j\theta_{g,t,c,1}},\ldots ,e^{j\theta_{g,t,c,K}}\right]}^{T}$. From this, the reflecting-time block of layer *c* is

(15) $$\begin{array}{c} X_{g,t,c}=\mathrm{diag}\left\{s_{g,t,c}\right\}Z_{g,t} \end{array}$$

By means of differential encoding processing, the reflecting-time transmission matrix of layer *c* can be expressed as

(16) $$\begin{array}{c} R_{g,t,c}=R_{g,t-1,c}X_{g,t,c} \end{array}$$

where $R_{g,t,c}$ need to satisfy the following conditions:

(1) If *m* is odd, the initial matrix of the 1st layer is $R_{g,0,1}=R_{1}I_{K}$; Conversely, when *m* is even, $R_{g,0,1}=R_{1}e^{j\frac{\pi }{4}}I_{K}$. For other layers (2≤c≤C), $R_{g,0,c}=R_{c}e^{j\frac{\pi }{4}}I_{K}$. Here, $R_{1}=2^{C-1}\sqrt{2^{\mathrm{mod}(m+1,2)}}$ (c=1) and $R_{c}=2^{C-c}\sqrt{2}$ (2≤c≤C) denote the magnitudes of constellation mapping for each layer.
(2) The same reflecting-time permutation matrix $Z_{g,t}$ is assigned across all layers. This assignment guarantees that, for $R_{g,t,c}$ in different layers, each column and row has only one non-zero value at the same position.

After superimposing all layers, according to [30], the final reflecting-time transmission matrix can be expressed as

(17) $$\begin{array}{c} {\bar{R}}_{g,t}=\sum_{c=1}^{C}R_{g,t,c} \end{array}$$

where each column and row of ${\bar{R}}_{g,t}$ has precisely one non-zero value, meaning that, at each instant, only one reflection mode is activated exactly once in *K* consecutive timeslots.

The differential encoding processing of the time–frequency transmission block is in accordance with Equation (3). As in Equation (5), the final fused two-dimensional matrix ${\bar{S}}_{g,t}\in C^{LK\times LK}$ can be formulated as

(18) $$\begin{array}{c} {\bar{S}}_{g,t}=F_{g,t}⊗{\bar{R}}_{g,t}=F_{g,t}⊗\left(\sum_{c=1}^{C}R_{g,t,c}\right) \end{array}$$

### 3.2. Receiver Detection

At the receiver, the frequency-domain signal matrix received in group *g*, time instant *t* can be expressed as

(19) $$\begin{array}{cc} Y_{g,t} & =H_{g}{\bar{S}}_{g,t}+V_{g,t}=\sum_{c=1}^{C}Y_{g,t,c}+V_{g,t} \end{array}$$

where $Y_{g,t,c}=H_{g}\left(F_{g,t}⊗R_{g,t,c}\right)$. Similarly, the zero-noise received matrix in group *g*, time instant t−1, layer *c* is $Y_{g,t-1,c}=H_{g}\left(F_{g,t-1}⊗R_{g,t-1,c}\right)$. According to Equations (12) and (13), $Y_{g,t,c}$ can also be expressed as

(20) $$\begin{array}{c} Y_{g,t,c}=Y_{g,t-1,c}\left(A_{g,t}⊗X_{g,t,c}\right) \end{array}$$

Substituting Equation (20) into Equation (19) yields

(21) $$\begin{array}{c} \left({\hat{A}}_{g,t},{\hat{X}}_{g,t,1},\ldots ,{\hat{X}}_{g,t,C}\right)\\ =\underset{\forall A_{g}\in \psi_{F},X_{g,c}\in \psi_{R_{c}}}{\arg\min}{∥Y_{g,t}-\sum_{c=1}^{C}Y_{g,t-1,c}\left(A_{g}⊗X_{g,c}\right)∥}_{F}^{2} \end{array}$$

(22) $$\begin{array}{cc} \; & Y_{g,t,c}=Y_{g,t-1,c}\left({\hat{A}}_{g,t}⊗{\hat{X}}_{g,t,c}\right) \end{array}$$

where $\psi_{R_{c}}$ is the set of all legitimate $X_{g,c}$ in layer *c*. Equation (21) fundamentally relies on a search over the finite alphabets of $A_{g}$ and the per-layer symbols $X_{g,c}$. The search space is extended to jointly cover the reflecting pattern $A_{g}$ and the set of per-layer symbol matrices $\left\{X_{g,1},\ldots ,X_{g,C}\right\}$, each from its corresponding constellation set $\psi_{R_{c}}$. Similarly to the PSK-aided DRFM system, once ${\hat{Z}}_{g,t}$ is known, the estimated layer symbol vector ${\hat{s}}_{g,t,c}$ is obtained by applying the inverse permutation ${\hat{Z}}_{g,t}^{T}$ to the vector of non-zero elements extracted from each per-layer block ${\hat{X}}_{g,t,c}$. Then, each layer’s vector ${\hat{s}}_{g,t,c}$ is first PSK-demapped, and the resulting bits from all *C* layers are then recombined according to the transmitter’s superposition rule to reconstruct the original high-order QAM symbol bits. When *m* is odd, for the first layer (c=1), the initial zero-noise received matrix is defined as $Y_{g,0,1}=\frac{Y_{g,0}R_{1}}{R_{1}+\sum_{a=2}^{C}R_{a}e^{j\frac{\pi }{4}}}$. For the subsequent layers with c∈{2,…,C}, the initial zero-noise received matrices are expressed as $Y_{g,0,c}=\frac{Y_{g,0}R_{c}e^{j\frac{\pi }{4}}}{R_{1}+\sum_{a=2}^{C}R_{a}e^{j\frac{\pi }{4}}}$. When *m* is even, for each layer with c=1,…,C, the initial zero-noise received matrix is set to $Y_{g,0,c}=\frac{Y_{g,0}R_{c}}{\sum_{a=1}^{C}R_{a}}$. In accordance with Equations (21) and (22), the receiver requires the previous t−1-th time–frequency permutation matrix ${\hat{A}}_{g,t-1}$ and the reflecting-time signal matrix ${\hat{X}}_{g,t-1,c}$ to determine the zero-noise received matrix $Y_{g,t-1,c}$ for the detection process in the current *t*-th block. Consequently, any amplitude errors in the detected signal may give rise to the issue of error accumulation and propagation.

## 4. Simulations and Analysis

In this section, we showcase the simulation results of the proposed DRFM system and the applicable high-order QAM modulation scheme. To demonstrate the BER performance of the proposed DRFM system, we compare DRFM with non-DRFM (NDRFM). In NDRFM, perfect CSI $H_{g,l}$ (i.e., perfect $h_{g,l,1}$, $H_{g,l,2}$, and $h_{g,l,d}$) is adopted for coherent detection. Notably, to ensure a fair comparison, the Kronecker product is incorporated into the operational framework of NDRFM as well. Under this premise, the spectral efficiency of NDRFM can be expressed as

(23) $$\begin{array}{c} \eta_{\mathrm{NDRFM}}=\frac{G(\log_{2}\left(L!\right)+K\log_{2}\left(K\right)+K\log_{2}\left(M\right))}{K(F+L_{cp})} \end{array}$$

To further verify the BER performance of the high-order QAM scheme suitable for DRFM, we incorporate two-amplitude APSK modulation [27] and four-amplitude APSK modulation [28] into the proposed DRFM system. Moreover, we implement the precoding normalized (PN) scheme cited in reference [29] within the DRFM framework, obtaining a PN-DRFM system based on star-QAM modulation for a comparative analysis. In the comparison, we consider an RIS with N=4 elements, each using 1-bit coding (${\left(\Phi_{k}\right)}_{nn}\in \left\{1,-1\right\}$), resulting in $2^{4}=16$ valid reflecting patterns. Following [19], K=2 patterns are selected via the stepwise depletion algorithm. The related simulation parameters are listed in Table 2. The SNR is $S/N_{0}=G/\sigma^{2}$. The carrier frequency is set as 5.9 GHz.

**Figure 5:**
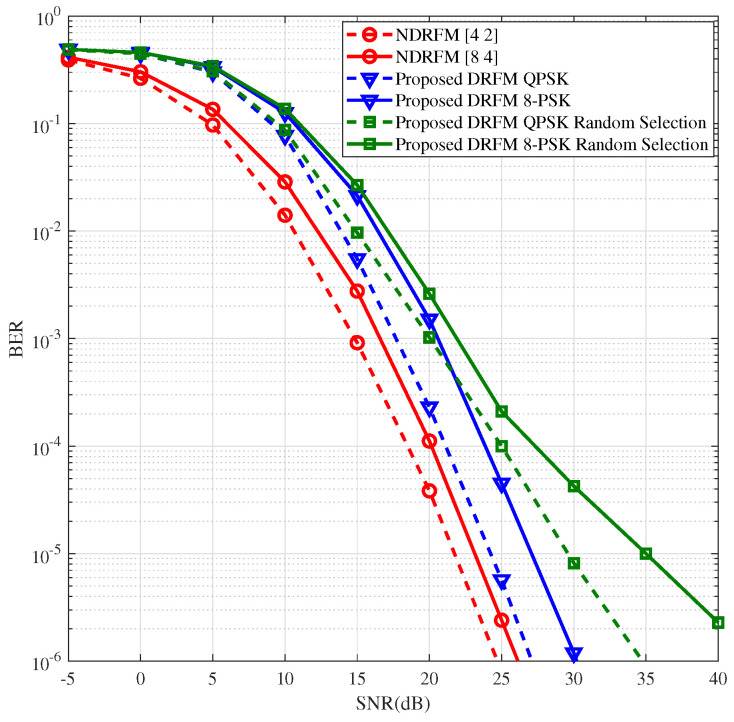
BER comparisons in RIS-assisted communication system with perfect CSI.

In Figure 5, we present and compare the BER performance of the proposed PSK-aided DRFM and NDRFM in RIS-based reflecting-frequency dual-domain IM schemes. For an SE of 1.2 bps/Hz, DRFM uses QPSK modulation, while NDRFM uses mixed QPSK and BPSK modulation. For an SE of 1.6 bps/Hz, DRFM employs 8-PSK modulation, and NDRFM adopts mixed 8-PSK and QPSK modulation. To ensure fairness, both systems employ ML detection. Figure 5 illustrates that, under the given simulation setups, DRFM is at a 3–4 dB disadvantage relative to NDRFM, while eliminating the receiving end’s need to acquire CSI. For further comparison, in Figure 5, we include the BER of DRFM with the reflecting pattern selection in [19] and contrast it with random selection, demonstrating the superiority of this reflecting pattern selection.

**Figure 6:**
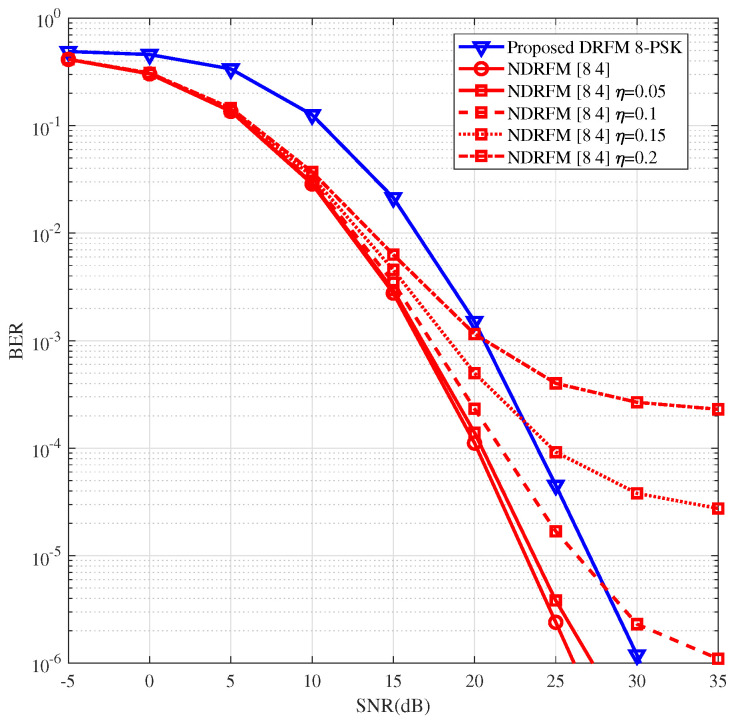
BER comparisons in RIS-assisted communication system with imperfect CSI.

Recall that, in the previous simulation, perfect CSI was assumed for NDRFM. However, NDRFM demands substantial resources for channel estimation, and perfect CSI is rarely available. To evaluate the impact of CSI estimation errors on performance degradation, in Figure 6, we further compare the proposed DRFM and NDRFM with imperfect CSI. From Figure 6, the performance of NDRFM deteriorates notably as the CSI estimation error η rises. When η=0.05, the performance gap between DRFM and NDRFM is small. When η≥0.1, in the high-SNR range, DRFM with coherent detection outperforms NDRFM, with NDRFM showing a significant floor effect.

**Figure 7:**
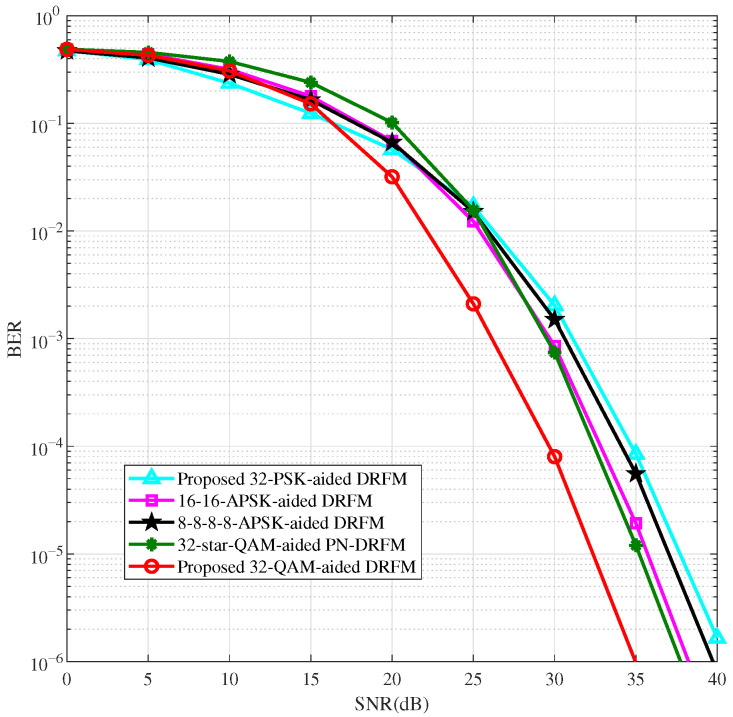
BER comparison of DRFM system with M=32.

**Figure 8:**
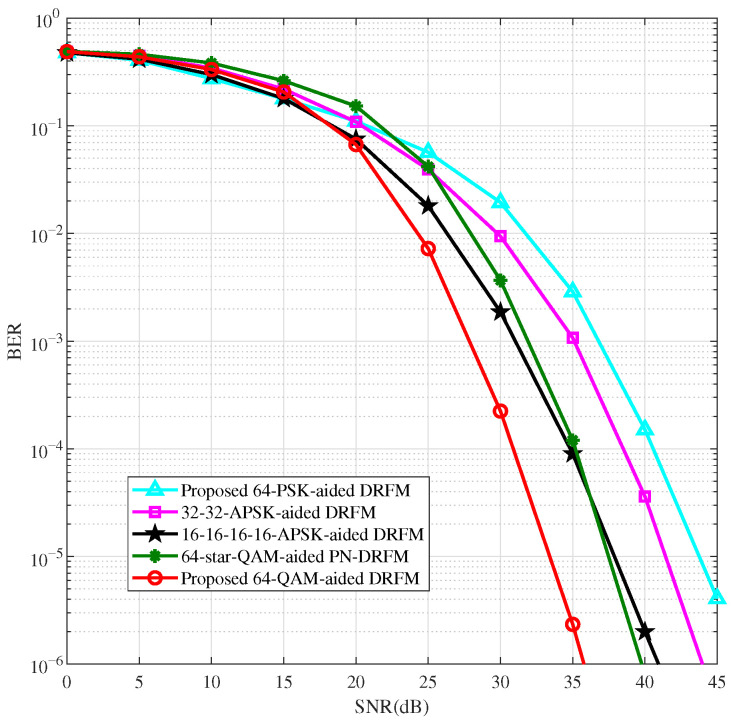
BER comparison of DRFM system with M=64.

To further validate the effectiveness and reliability of the high-order QAM modulation scheme, we apply the proposed QAM and existing non-constant-modulus modulation schemes [27,28,29] to DRFM for performance comparison. Figure 7 and Figure 8 display the BER performance comparison of DRFM using QAM, multi-amplitude APSK (two-amplitude and four-amplitude APSK), PSK, and PN-DRFM using star-QAM for M=32 and M=64 modulations, with spectral efficiencies of 2.4 bit/s/Hz and 2.8 bit/s/Hz, respectively. Ref. [27] states that the two amplitudes of two-amplitude APSK are $\left(r_{L},r_{H}\right)=\left(0.632,1.265\right)$. Refs. [28,29] indicate that the optimal amplitude ratios of four-amplitude APSK and star-QAM are identical. For M=32 and M=64, their optimal amplitude ratios are 1.5 and 1.35, respectively.

As shown in Figure 7 and Figure 8, in the high-SNR region, the BER performance of the proposed QAM-aided DRFM system is better than that of the star-QAM-aided PN-DRFM system and the multi-amplitude APSK and PSK-aided DRFM systems. This is due to the relatively uniform distribution of QAM constellation points, leading to the highest utilization of spatial degrees of freedom. Among the other modulation methods, the utilization of spatial degrees of freedom, from high to low, follows the order of star-QAM, multi-amplitude APSK, and PSK. As shown in Figure 7, the M=32 QAM intersects with PSK at an SNR of 16 dB. The intersection points of several other modulation methods with PSK are between approximately 15 dB and 23 dB. At BER = $10^{-5}$, when compared to the DRFM systems employing PSK, two-amplitude APSK (16-16-APSK, where two concentric rings each contain 16 phase points), four-amplitude APSK (8-8-8-8-APSK, where four rings each contain 8 points), and the PN-DRFM system using star-QAM, the QAM-based DRFM system achieves performance gains of approximately 6 dB, 3.5 dB, 5 dB, and 3 dB, respectively. In Figure 8, where M=64 and at a BER of $10^{-5}$, relative to the DRFM systems using PSK, two-amplitude APSK (32-32-APSK), and four-amplitude APSK (16-16-16-16-APSK), and the PN-DRFM system with star-QAM, the QAM-based DRFM system attains performance gains of around 10 dB, 8 dB, 4.5 dB, and 4 dB, respectively.

**Figure 9:**
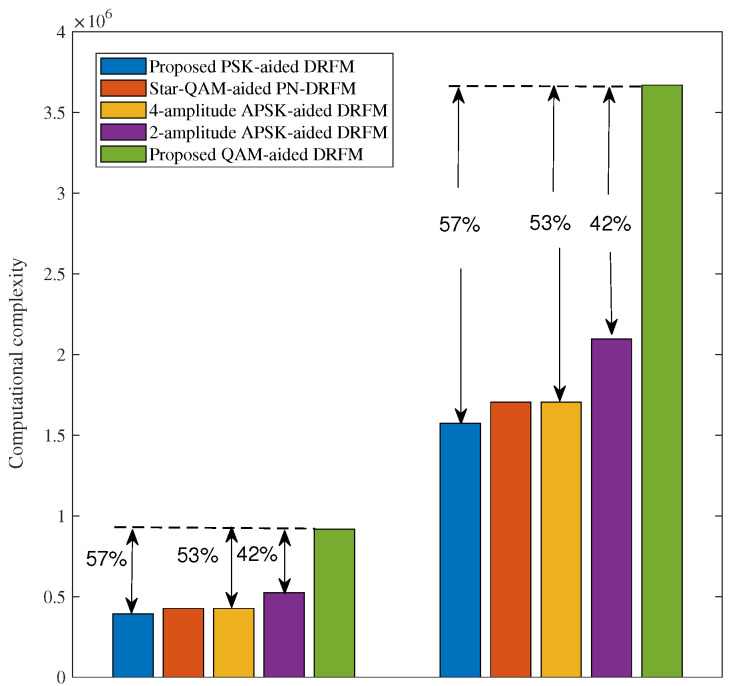
Computational complexity comparison of DRFM system with M=32 and M=64.

Table 3 compares the complexity of different constellation modulations within one carrier group of DRFM—specifically, the number of real-valued multiplication operations required. Here, *B* denotes the number of bits transmitted in one reflecting-time–frequency block. As shown in Table 3, $Y_{g,t-1,c}\left(A_{g}⊗X_{g,c}\right)$ necessitates $4L^{2}N_{r}K$ real-valued multiplication operations. The 2-norm operation demands $2L^{2}N_{r}K$ real-valued multiplication operations. The QAM scheme presented in this paper partitions the QAM constellation into *C*-layer differential transmissions. Consequently, the number of real-valued multiplications grows by a factor of (2C+1)/3 relative to PSK. Thus, it exhibits the highest computational complexity. Figure 9 depicts the complexity comparison under specific settings: K=L=2, $N_{r}=2$, and M=32 and 64. Relative to PSK, star-QAM, four-amplitude APSK, and two-amplitude APSK, the complexity of the QAM-based DRFM system increases by approximately 57%, 53%, 53%, and 42%, respectively. Nevertheless, as is evident from Figure 8, when M=64 and $BER=10^{-6}$, compared to the DRFM systems using PSK, two-amplitude APSK, and four-amplitude APSK, and the PN-DRFM system using star-QAM, the proposed QAM-aided DRFM system achieves performance gains of around 10 dB, 8 dB, 4.5 dB, and 4 dB, respectively.

## 5. Conclusions

In this paper, we generalize DRM from single-carrier to multi-carrier scenarios and propose the DRFM system for RIS-based communications. In DRFM, the transmitter integrates the differentially coded reflecting-time block and the time–frequency block using the Kronecker product. At the receiver, differential detection allows operation without acquiring CSI. Moreover, a non-constant QAM constellation scheme is explored for the DRFM system. The transmitted QAM constellation is encoded and transmitted hierarchically and differentially via the composite phase shift method. Numerical simulations have been carried out to validate the advantages of the DRFM system and the applicable QAM constellation modulation scheme in terms of the BER. The performance was evaluated under ideal synchronization as a baseline to validate the core concept. Future work will investigate robustness to synchronization errors and extend this CSI-free paradigm to multi-user scenarios, leveraging classical RIS phase optimization techniques.

## Figures and Tables

**Table 1:** List of acronyms.

Acronym	Full Form
RIS	Reconfigurable Intelligent Surface
IM	Index Modulation
CSI	Channel State Information
DRM	Differential Reflecting Modulation
DRFM	Differential Reflecting Frequency Modulation
QAM	Quadrature Amplitude Modulation
APSK	Amplitude Phase Shift Keying
DSM	Differential Spatial Modulation
BER	Bit Error Rate
OFDM	Orthogonal Frequency Division Multiplexing
SIMO	Single-Input Multiple-Output
Tx	Transmitter
Rx	Receiver
PSK	Phase Shift Keying
ML	Maximum-Likelihood
NDRFM	Non-DRFM
PN-DRFM	Precoding-Normalized DRFM

**Table 2:** The definitions and values corresponding to the DRFM system.

Notation	Definition	Value
*M*	Modulation orders	4/8/32/64
$L_{cp}$	Cyclic prefix	8
*F*	Number of subcarriers	32
*K*	Number of reflecting patterns	2
$N_{r}$	Number of receive antennas	2
*T*	Number of transmitted blocks	20
*L*	Number of subcarriers per group	2

**Table 3:** Comparison of the complexity of DRFM with different modulations.

Modulation Methods	Receiver Detector	Number of Real-Valued Multiplications
Proposed PSK	${∥Y_{g,t}-Y_{g,t-1}\left(A_{g}⊗X_{g}\right)∥}_{F}^{2}$	$\left(6L^{2}N_{r}K\right)2^{B}$
Proposed QAM	${∥Y_{g,t}-\sum_{c=1}^{C}Y_{g,t-1,c}\left(A_{g}⊗X_{g,c}\right)∥}_{F}^{2}$	$\left(\left(4C\;+\;2\right)L^{2}N_{r}K\right)2^{B}$
Two-amplitude APSK [27]	${∥\begin{array}{cc} & Y_{g,t}-Y_{g,t-1}{\hat{M}}_{g,t-1}^{-1}\\ & \;\Lambda_{g,t}{\hat{M}}_{g,t-1}\left(A_{g}⊗X_{g}\right) \end{array}∥}_{F}^{2}$	$\left(6L^{2}N_{r}K\;+\;8LK\right)2^{B}$
Four-amplitude APSK [28]	${∥Y_{g,t}-Y_{g,t-1}\Lambda_{g,t}\left(A_{g}⊗X_{g}\right)∥}_{F}^{2}$	$\left(6L^{2}N_{r}K\;+\;2LK\right)2^{B}$
Star-QAM of PN-DRFM [29]	${∥Y_{g,t}-Y_{g,t-1}P_{g,t-1}\left(A_{g}⊗X_{g}\right)∥}_{F}^{2}$	$\left(6L^{2}N_{r}K\;+\;2LK\right)2^{B}$

## Data Availability

The data presented in this study are available on request from the first author.
